# Studying Chromatin Epigenetics with Fluorescence Microscopy

**DOI:** 10.3390/ijms23168988

**Published:** 2022-08-12

**Authors:** Afanasii I. Stepanov, Zlata V. Besedovskaia, Maria A. Moshareva, Konstantin A. Lukyanov, Lidia V. Putlyaeva

**Affiliations:** 1 Center of Molecular and Cellular Biology, Skolkovo Institute of Science and Technology, Bolshoi Blvd. 30, Bld. 1, 121205 Moscow, Russia; 2 Shemyakin-Ovchinnikov Institute of Bioorganic Chemistry, Russian Academy of Sciences, Miklukho-Maklay St. 16/10, 117997 Moscow, Russia

**Keywords:** epigenetics, histone modification, fluorescent proteins, genetically encoded probes

## Abstract

Epigenetic modifications of histones (methylation, acetylation, phosphorylation, etc.) are of great importance in determining the functional state of chromatin. Changes in epigenome underlay all basic biological processes, such as cell division, differentiation, aging, and cancerous transformation. Post-translational histone modifications are mainly studied by immunoprecipitation with high-throughput sequencing (ChIP-Seq). It enables an accurate profiling of target modifications along the genome, but suffers from the high cost of analysis and the inability to work with living cells. Fluorescence microscopy represents an attractive complementary approach to characterize epigenetics. It can be applied to both live and fixed cells, easily compatible with high-throughput screening, and provide access to rich spatial information down to the single cell level. In this review, we discuss various fluorescent probes for histone modification detection. Various types of live-cell imaging epigenetic sensors suitable for conventional as well as super-resolution fluorescence microscopy are described. We also focus on problems and future perspectives in the development of fluorescent probes for epigenetics.

## 1. Introduction

Epigenetic modifications provide genome plasticity and enable cells to express a specific set of genes. They include DNA methylation, diverse modifications of histone proteins and also the modulation of gene expression in cis by coating DNA with non-coding RNA (for example, X-chromosome inactivation). Histones are basic proteins that form a structural scaffold in chromatin and undergo a significant degree of post-translational modifications mainly in their N-terminal tails. These changes serve as recognition sites for a variety of chromatin-binding proteins, as well as regulating steric access to DNA for replication and transcription machinery. Understanding histone modification dynamics is thus critical for deciphering the mechanisms of epigenetic regulation.

One of the major mechanisms for this regulation is post-translational modifications—methylation, acetylation, phosphorylation, and some others—that are abundant mainly at the N-terminal regions of histone proteins, especially histones H3 and H4. There are protein domains that are responsible for introducing modifications to histone molecules (“writer” enzymes) and removing these modifications (“eraser” enzymes). In addition to changing the physicochemical properties of histones [1], modifications act as signaling molecules that mark chromatin with certain properties. Histone modification “reader” domains (HMRD) play a key role in the interpretation of the histone code by the cell. HMRDs attract proteins with specific activities required at a given chromatin locus [2]. The insertion, recognition and removal of histone modifications is dynamic and adjustable at many levels. It is important to note that some patterns of histone modifications can be epigenetically inherited by daughter cells [3].

Despite their critical roles in regulating chromatin architecture, it is unknown how epigenetic modifications are dynamically coordinated with chromatin remodeling. Therefore, the development of sensitive and precise methods for epigenome tracking, ideally within intact living cells is critical. Monoclonal antibodies that specifically bind with various post-translational modifications of histones play an important methodological role in the study of this problem [4]. Such antibodies allowed obtaining basic information about the functional significance of histone modifications using the ChIP-Seq method, which includes chromatin immunoprecipitation and subsequent high-throughput DNA sequencing [5,6]. In recent years, an extraordinary interest has emerged in the study of chromatin at the level of single cells. Methods for analyzing DNA methylation, histone modifications, DNA accessibility and chromatin conformation based on high-throughput DNA sequencing from single cells have been proposed [3,7]. Such approaches allow us to identify cellular plasticity and heterogeneity, which is especially important when studying stem cells and carcinogenesis. In combination with the sequencing of genomes and transcriptomes of single cells, the characterization of chromatin organization provides important results on the intratumoral heterogeneity of cancer cells and its role in resistance to chemotherapy and clonal evolution [8]. The main disadvantages of these methods are the high complexity and cost of analysis, as well as the loss of information about the spatial location of the studied cells.

Another way to characterize individual cells can be fluorescence microscopy, due to its relative simplicity and the rich spatial information provided. In this review, we discuss fluorescence imaging strategies for detecting histone modifications in both fixed and living samples.

## 2. Histone Modification Imaging

### 2.1. Immunofluorescence in Histone Modification Imaging

Immunofluorescence is a classical approach to image various epitopes, including modified histones, using specific antibodies [9]. In this paper, we consider recent and most profound advances in epigenetics using immunofluorescent staining.

**Figure 1:**
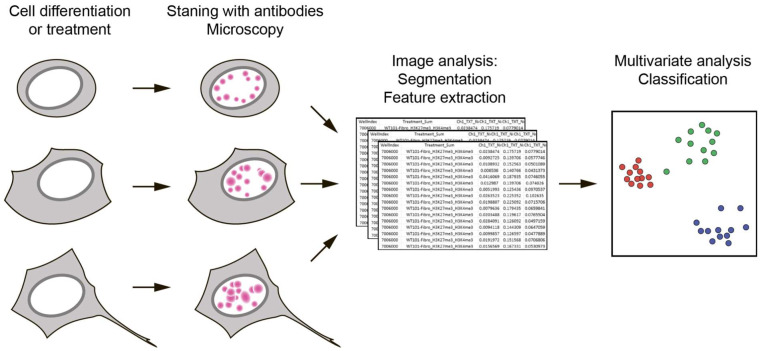
Schematic outline of MIEL. Cells of interest (e.g., cells at different stages of differentiation and cells after drug treatment) are stained using antibodies against target epigenetic modification(s). The obtained intranuclear patterns (landscapes) of distribution of histone modifications undergo computer analysis (texture features extraction). Cells are compared and classified using the multiparametric Euclidean distances between them.

In 2019, Terskikh and coworkers proposed a new method called Microscopic Imaging of Epigenetic Landscapes (MIEL) [10]. At the first stage, immunocytochemical staining of fixed cells with antibodies specific to the selected histone modifications and their fluorescence microscopy was performed. This makes it possible to visualize intranuclear patterns (landscapes) of fluorescent signals characteristic of each type of modification in these cells. At the second stage, the obtained arrays of images of individual nuclei were subjected to mathematical analysis. Multivariate image analysis and machine learning [11] allowed one to identify several hundred features of these landscapes and compare different cell samples with each other in a virtual multidimensional space using multidimensional scaling (MDS) (Figure 1).

Terskikh and co-authors have shown that MIEL allows: (1) to identify epigenetic landscapes characteristic of various cell lines; (2) to detect changes in chromatin under the influence of various drugs, while drugs of the same mechanism of action lead to similar changes in the landscapes of histone modifications; (3) monitor chromatin changes during cell differentiation (for example, reprogramming human fibroblasts into iPSCs and further differentiation into neuronal cells). High-throughput sequencing (RNA-Seq) confirmed a high degree of correlation between MIEL cell comparisons and standard transcriptome analysis. Thus, MIEL is a promising tool for revealing differences in the epigenetic state of cells in various biological models, as well as for the high-throughput screening of drugs [10].

In 2020, Hayashi-Takanaka et al. described the method for providing detailed information on histone modifications using multicolor immunofluorescence-based single cell analysis [12]. They found that approximately 400–500 images of nuclei using lenses with 40× dry objective lens with NA 0.75–1.25 are usually sufficient to obtain feasible and reproducible results for analysis. It was demonstrated that the levels of H4K5ac and H3K4 methylation (active histone marks) were increased during the S phase, and in contrast, levels of H4K20me1 (repressive modification) increased during the G2 phase [12].

Recently, Takei et al. developed the new approach to characterize nuclear architecture using a combination of DNA seqFISH+, multiplexed IF and RNA seqFISH methods [13]. The group defined 12 major clusters in single nuclei at 1 Mb resolution that form physically distinct regions according to different combinatorial marks. Additionally, the appearance of transcription active sites at the surface of nuclei and independence of chromatin states from cell-cycle phases were revealed. Additionally, clonal analysis experiments showed that most of chromatin features are inherited over at least three to four generations.

Another straightforward approach was published in February 2022—Rong Fan group developed the method that they called Spatial-CUT&Tag [14]. It exploited two serial sets of barcoding to achieve a two-dimensional map of tissue “pixels” in combination with pA-Tn5 transposition and next-generation sequencing. Mapping the clusters of histone modifications (H3K27me3, H3K4me3, etc.) back to spatial locations identified spatially distinct patterns that agreed with the tissue histology in mouse embryo brain and olfactory bulb. In the future, Spatial-CUT&Tag may become an unbiased next-generation-sequencing-based approach by using a serpentine microfluidic channel or increasing the number of barcodes to increase the mapping area.

However, immunofluorescent analysis of chromatin is limited to fixed samples only. It cannot be used to track the dynamics of epigenetic changes in live cells, for example, in the course of cell differentiation or drug treatment. To fill this gap, a number of genetically encoded epigenetic probes were developed as discussed below.

### 2.2. Genetically Encoded Probes for Live-Cell Imaging of Histone Modifications

#### 2.2.1. Antibody-Derived Probes

**Figure 2:**
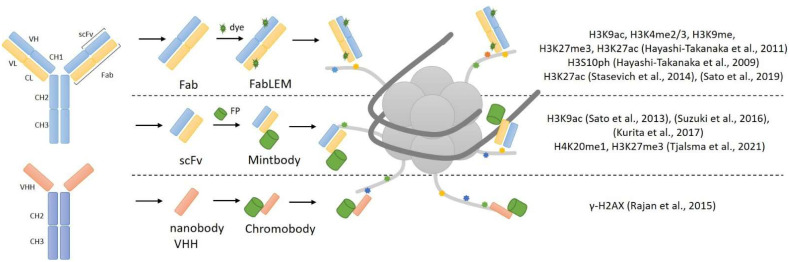
Schematic representation of the antibody-derived probes for histone modification imaging [16,17,18,19,20,21,22,23,24,26].

Antibody immunostaining is currently the best and unique method for imaging proteins and different targets, and the potential use of antibodies for live-cell imaging is an ideal option. Histone modifications are good targets for synthesized antibodies, but whole antibodies cannot be used within living cells. Immunoglobulin G (IgG), used for immunostaining, consists of four chains: two light (L) and two heavy (H). These chains form the constant (Fc) and antigen-binding (Fab) region, which forms the complementary-determining region (CDR). Because IgG’s size of 150 kDa prevents it from passing through nuclear pores, reduced variants of antibodies have been developed. The monovalent antibody fragment (Fab) has a size of 50 kDa and has the ability to pass through nuclear pores [15]. One other version of the small-size antibody is a single-chain variable fragment (scFv) approximately 25 kDa and consists solely of VL and VH chains. In contrast to full-sized antibodies, scFv can easily be expressed intracellularly from any suitable vector [15]. The general structure of antibody-derived reporters for histone modification imaging is presented in Figure 2.

The first approach for monitoring the distribution of histone modification in live cells, based on fluorescently labeled Fab fragments, was invented in 2011 and called FabLEM [16]. Primarily, specific antibodies to certain modifications of histones were synthesized, and based on these antibodies, Fab fragments were made (by protease digestion) and then conjugated with dyes. As mentioned above, Fab fragments can penetrate nuclear pores and have been used in living cells and mouse embryos [16]. Hayashi-Takanaka et al. used Fab fragments for the detection of H3K9ac, H3K4me2/3, H3K9me2, H3K27me3 and H3K27ac histone modifications in their work. They injected specific Fab fragments to histone modification into live cells, and then cells were fixed and immunostained with an antibody to the same histone modification. Colocalization analysis showed that Fab fragments specifically bind to tail modifications. They also showed that the binding is transient and does not block access to histone modifications for other compounds, so the Fab fragments were not toxic to living cells and embryos and did not affect their growth and development.

Additionally, Fab fragments were used in the study of H3S10 histone modification in living cells [17], the effect of H3K27ac levels on the activity of RNA polymerase [18] and the development of zebrafish embryos [19].

The development of genetically encoded probes based on antibodies was the next step in the live-cell imaging of post-transcriptional modifications of histones. Yuko Sato et al. [20] created a genetically encoded system for tracking histone modifications. They made genetically encoded modification-specific intracellular antibodies (mintbodies) that consist of a single-chain variable fragment (scFv) fused with a green fluorescent protein (GFP). scFv specifically recognizes and binds to histone modification; it has a small size and can be easily expressed from a genetic cassette with a fluorescent protein. The key advantage of mintbodies is their ability to prevent aggregation of scFvs because they are not secreted outside of the cells and instead remain permanently inside the cell nucleus. This technique has been used to track H3K9ac in living cells and living organisms [20]. After loading genetic probes into live cells, mintbodies were expressed in the nucleus and specifically bound to H3K9ac. Mintbodies detected changes in H3K9ac levels after treatment with the histone deacetylase inhibitor trichostatin. They also showed that mintbody has no significant effects on the growth and development of living cells and organisms, because mintbodies, as Fab fragments [16], bind transiently and do not block access to histone modifications [20]. This was demonstrated in an experiment involving a transgenic fly line expressing H3K9ac-recognizing mintbody during embryogenesis.

Additionally, H3K9ac mintbody was used in Xenopus laevis [21] and in plant cells [22]. Along with H3K9ac, specific mintbody to H4K20me1 [23] and H3K27me3 histone modifications were also published [24]. To date, only some variants of mintbodies have been invented (for H3K9ac, H3K27me3 and H4K20me1), since using genetically encoded mintbodies is more convenient than Fabs [25].

As mentioned earlier, scFvs are truncated variants of full-length antibodies that only contain the variable domains of heavy (VH) and light chains (VL) [15]. Camelidae sdAbs were used to create nanobodies, which are antigen-binding bodies consisting solely of a variable domain of the heavy chain (VHH) with a size of around 13 kDa [15]. Malini Rajan et al. [26] used in their work specific nanobodies to γ-H2AX (which they called ‘chromobodies’) in a fusion with a green fluorescent protein. With the help of immunization, they created an antibody to γ-H2AX, and then the VHH domain was taken from it and cloned into a vector with GFP. They assessed the localization of the nanobody at the sites of DNA damage. Other epitopes were recognized and bound by these nanobodies, indicating that their specificity is incorrect.

#### 2.2.2. FRET Sensors

**Figure 3:**
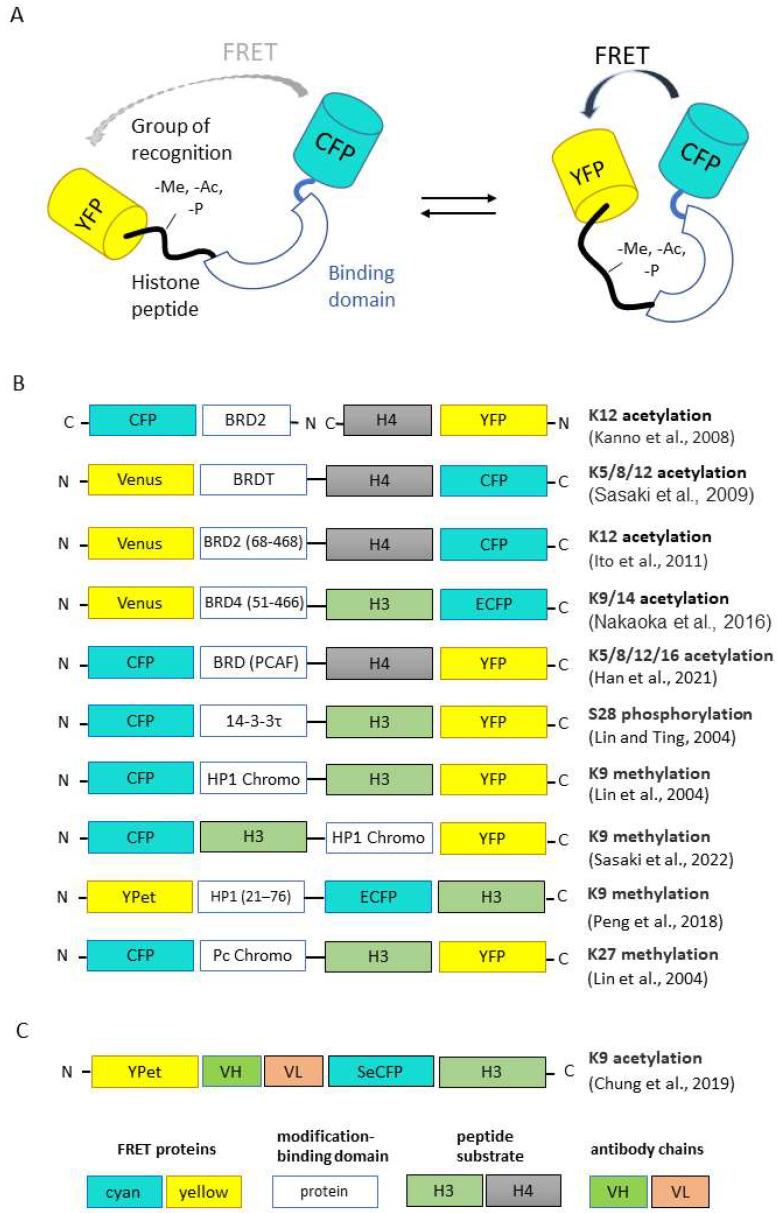
Genetically encoded FRET sensors for live-cell imaging. (**A**) General design of FRET sensors. (**B**,**C**) Domain structures of FP-based and scFv-based FRET sensors, respectively. Here, and in analogous schemes, below protein parts are not to scale [36,37,38,39,40,41,42,43,44,45].

Basically, genetically encoded Förster Resonance Energy Transfer (FRET) sensors consist of two fluorescent proteins flanking a recognition domain and forming a single polypeptide chain (Figure 3A). Ligand binding to the recognition domain results in a change in the distance between fluorescent proteins (FPs) and their relative orientation, and thus a change in the FRET efficiency. The first FRET sensor was designed in the Roger Tsien lab in 1997 to detect Ca^2+^ [27]. After that, dozens of genetically encoded FRET sensors were developed for a wide range of stimuli, including ions, small molecules, enzyme activities and membrane potential [28]. Along with the ability of monitoring the spatiotemporal distribution of fluorescent signals in real time, bulk FRET-based techniques have some limitations in epigenetic studies. The most important drawback is that heterogeneity of nucleosomes, including intermediate conformational states and different compositions of epigenetic marks, becomes hidden at the ensemble level. To solve this problem, single molecule FRET (smFRET) can be used. As smFRET analyzes one nucleosome at a time, it clearly distinguishes different species without averaging [29,30]. smFRET was successfully used to study spontaneous nucleosome core fluctuation [31], transcription elongation through the nucleosomes by Pol II [32], structural intermediates during salt-dependent nucleosome dissociation [33], etc. smFRET also enabled the characterization of the key role protein complex FACT in nucleosome-stabilizing activity [34] and the ATP-independent modification of the nucleosomal structure that results in the reversible uncoiling of DNA [35].

The schematic representation of FRET-based epigenetic sensors described below is presented in Figure 3B. Chi-Wang Lin and Alice Ting developed the first FRET-based reporter specific for phosphorylated Ser28 of histone H3 [36].To create a sensing core, the authors fused the N-terminal fragment of histone H3 (positions 1–30, which includes the target Ser28) with a phosphoserine/threonine-binding domain (protein 14-3-3t, 232 aa). Connected by a flexible linker, these parts changed their relative orientation upon the phosphorylation of Ser28. The sensing core was inserted between cyan and yellow fluorescent proteins consisting of a classical CFP-YFP FRET-pair. Thus, Ser28 phosphorylation-induced conformational changes led to the increase in FRET efficiency between CFP and YFP. This group also published the sensors recognizing H3K9me3 and H3K27me3 with replacing phosphoserine/threonine-binding module to methyllysine-binding domain HP1 (for H3K9me3 specificity) or Polycomb (Pc) chromodomain (residues 21−78) [37].

The first FRET-based sensor specific for acetylation was published by Kanno et al. [38]. The researchers used bromodomain protein Brd2, which can interact selectively with acetylated lysine 12 on histone H4, fused with cyan fluorescent protein (CFP), as a donor for FRET analysis, and the fuse of histone H4 and YFP protein as acceptor. Then, the group of Minoru Yoshida published a set of FRET-based indicators known as Histac. The response of the first Histac indicator reflects changes in the acetylation state of both K5 and K8 in histone H4 [39], and the region of BRDT consisted of two bromodomains was used as the acetylation-binding module. The next variant of the sensor, Histac-K12, was designed by Ito et al. with the implementation of BRD2 bromodomain (68–468 aa), which specifically recognizes histone H4K12 acetylation [40]. Compared to [38], Ito et al. used only the bromodomain BRD2 and improved the linker between BRD2 and CFP. Using Histac-K12, the authors showed that the level of H4K12 acetylation was maintained during mitosis, but H4K5/K8 acetylation in mitotic chromosomes decreased. Later, the same group used different type of recognizing unit, namely the bromodomain fragment of “reader” protein BRD4 (residues 51–466) [41]. This FRET-based probe can specifically respond to the acetylation of H3K9/K14 and can be used for the evaluation of BRD4 inhibitors in living cells [41].

Peng et al. created a FRET biosensor to visualize the histone H3 Lys-9 trimethylation (H3K9me3) dynamics in single live cells [42]. When H3K9 is trimethylated, the chromodomain of heterochromatin protein 1 (HP1) can bind to H3K9me3, resulting in a strong FRET signal. The biosensor revealed that the decrease in H3K9me3 occurs in the G2 phase before global chromatin reorganization and nuclear envelope dissolution. During cell cycles, an anti-correlation between H3K9me3 and H3S10p dynamics was also demonstrated, with H3S10p promoting H3K9me3 depletion at the onset of mitosis. One other FRET sensor with sensitivity to H3K9me3 was created quite recently by Sasaki et al. [43]. The new FRET-based probe named Hismet-HP1αCD also based on chromodomain of HP1α as a recognition domain and can be used for evaluating the inhibitors of histone methyltransferases or histone demethylases in living cells. The authors also showed the significant increase in FRET emission ratio from G2 to prophase and then a stepwise decrease from prometaphase to anaphase, which indicates the increase in H3K9me3 during mitosis.

Recently, Han et al. proposed a new variation of the sensor for histone H4 acetylation state detection [44]. The authors used a human P300/CBP-associated factor (PCAF) bromodomain (BRD) as binding domain instead of BRDT or BRD-2 published by Sasaki et al. [39] as a detection unit for the acetylation of histone H4 lysines. In this new sensor, the BRD domain was fused with the substrate sequence with a length ranging from 1 to 30 amino acids at the N-terminal of histone H4. They established that lysine residues at positions 5, 8, 12 and 16 of the H4 substrate sequence were all acetylated in the presence of histone acetyltransferases. 

Chung et al. proposed to use an intrabody-based FRET probe, which represents the combination of FRET methodology with previously described acetyl H3K9-specific scFv (the so-called mintbodies [20]). The authors noticed that modification-binding proteins are not specific to a single modification and could bind several modification sites with low binding affinity (K_d_ 1–200 μM for chromodomains and 3–300 μM for bromodomains). Using the intrabody as a sensor and a FRET fluorescent–protein pair as reporters, the authors took advantages of both methods and could monitor histone-modification levels by ratiometric FRET quantification in living cells. The authors showed that the lack of the H3 tail in the probe had no significant effect on FRET response, indicating that the probe binds to endogenous acetylated histone H3K9 rather than the H3 sequence within the probe to increase its FRET efficiency [45]. Another modification of the FRET-based probe was proposed by Ghadiali et al. [46]. They invented the in vitro p300 HAT (histone acetyltransferase) activity sensor based on the formation of a quantum dot/peptide immunocomplex in the presence of acetyl-CoA. QDot–dye energy transfer occurs due to the binding of acetylated substrate peptide, based on the N-tail of histone H4/QDot complex and acceptor-dye-labeled antibody anti-H4K6 acetyl with acetyl-CoA and p300 HAT.

#### 2.2.3. BiFC-Sensors

Despite its certain advantages, FRET experiments are quite challenging to conduct due to the fact that FRET is dependent on many factors, such as close proximity, large amounts and certain stoichiometric ratios of fusion proteins in order to obtain valid data. The process of measuring and quantifying the FRET results is also difficult [47].

Another valuable method for imaging the protein–protein interactions in general and epigenetic landscape dynamics specifically is bimolecular fluorescence complementation (BiFC). The method itself is based on splitting a single fluorescent protein into two parts and attaching the parts to the proteins thought to interact within cells. The split fragments themselves lack fluorescence unless they are brought in close enough proximity to become activated [48].

A good example of the method application is the work by Vincenz and Kerppola who utilized the BiFC assay to look into the role of the conserved regions of chromatin-binding CBX proteins in their recruitment to certain chromatin regions alongside with the significance of the H3K27me3 modification in this process [49].

CBX is a part of the polycomb repressive complex 1 (PRC1) and has the ability to bind H3K27me3 [50]. It was suggested that the recruitment of the PRC1 complex to the chromatin region is mediated via the trimethylation of H3K27 by another repressive complex (PRC2) possessing methyltransferase activity [51,52].

To test this theory, several probes were produced containing each of the CBX family members and the first fluorescent protein fragment. The second part of the fluorescent protein was fused to the histone H3 isoforms. Strikingly, the results provided by the quantitative BiFC assay showed that CBX recruitment to the chromatin regions was mediated strictly by non-conserved domains of the proteins. Interestingly, the PRC1 recruitment dependence on the PRC2 activity was not confirmed. It was clearly shown that H3K27 trimethylation was not required for CBX-chromatin interaction in live cells. Moreover, chromatin-bound CBX proteins did not exhibit colocalization with the H3K27me3 modification outside the inactive X.

In a more recent study, a similar approach was used by Sekar et al. [53]. The authors took advantage of luciferase bioluminescence to produce specific sensors for the split-Renilla luciferase complementation system. Their aim was to image methylation at certain sites on H3 protein N-tails, namely H3K9 and H3K27.

In the designed sensors that activate themselves upon the methylation of either H3K9 or H3K27, specific substrate domains derived from H3 histone proteins are accompanied by chromodomains either from Suv39H1 protein or from HP1 protein. These units are inserted between the N- and C-terminal domain of the split-Renilla luciferase protein (RLuc8.6). Upon the binding of the sensor to the methylated histone tails, the optimal complementation of the luciferase is achieved, thus reconstructing its enzymatic activity and activating its bioluminescence properties. The efficacy of the sensor was evaluated in the experiment where cells stably expressing the probe were treated with methyltransferase inhibitors (Bix01294 and UNC0638). According to the obtained results, a concentration-dependent decrease in luciferase bioluminescence was observed.

Another interesting trait of this exact research was the successful usage of the novel sensor in vivo. The authors were able to assess the histone methylation landscape in mice carrying tumor xenografts derived from the sensor-expressing tumor cells. They further tested the sensor in response to the intratumoral injection of Bix01294 and, as expected, observed a significant drop in luciferase complementation levels. Such promising results make this sensor a good candidate for preclinical utilization.

Another group of researchers focused their efforts on the direct detection of changes in pericentromeric H3K9me3 levels and studied the dynamics of these changes upon drug treatment [54]. Centromeric mouse major satellites are areas of constitutive heterochromatin highly enriched in H3K9me3 [55] and involved in a number of cancers and other pathological processes [56,57], which makes them valuable targets for research. The BiFC-based Bimolecular Anchor Detector (BiAD) sensors were engineered by fusing a Zinc-finger anchor module (for DNA sequence recognition) and a HP1β chromo domain detector module (for H3K9me recognition) to each of the split non-fluorescent fragments of the mVenus fluorescent protein. These modules simultaneously bind to the specific DNA area and the H3K9me3 modification and, being brought in close proximity, allow for the reconstitution and activation of the fluorescent protein. The probes proved themselves highly sensitive by accurately detecting the changes in site-specific levels of H3K9me3 upon changes in the activity of methyltransferases involved in introducing pericentromeric H3K9me3 modifications.

**Figure 4:**
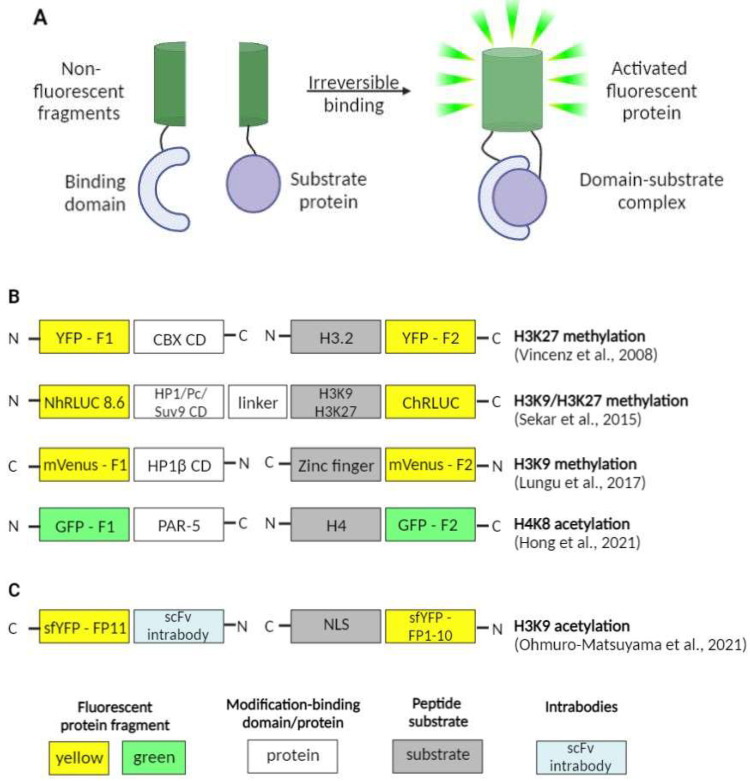
BiFC sensors for live-cell imaging. (**A**) General design of BiFC sensors. (**B**,**C**) Domain structures of FP-based and scFv-based BiFC sensors, respectively [49,53,54,58,63].

In a very recent work, Ohmuro-Matsuyama et al. addressed the issue of visualization of histone acetylation marks and the effects of histone deacetylase (HDAC) inhibitors [58]. They focused on the detection of the H3K9ac modification, a mark that is found to be involved in diseases as serious as Alzheimer’s disease [59], autism spectrum disorder [60] as well as several cancers [61,62]. For that, they used a split-YFP system, in which the fluorescent protein (sfYFP) was split into two uneven fragments. The smaller one was fused to the NLS (NLS-FP1-10), and the bigger one was brought together with the scFv intrabody (scFv-FP11). Intrabodies are stable antibodies that are able to retain their structures even in the cytoplasm and nucleus. When both of the probes are co-expressed in a live cell; the one with the NLS uses the nuclear import machinery and becomes accumulated in the nucleus. The nuclear levels of the second probe depend directly on the levels of H3K9ac. Additionally, the more abundant it becomes in the nucleus, the more scFv-FP11–NLS-FP1-10-binding events occur, leading to sfYFP reconstitution and increase in fluorescence signal, which allows for the detection and evaluation of HDAC inhibitor activity (Figure 4).

Another interesting work, also issued this year, tackled the issue of gut-governed behaviors in *Caenorhabditis elegans* [63]. The researchers not only showed that H4K8ac modification in the germline of *C. elegans* is essential for bacterial aversion behavior itself as well as for the transmission of such kind of behavior onto the next generation, but also that PAR-5 protein (a member of 14-3-3 chaperone protein family) is an important H4K8ac-interacting partner and may be involved in its regulation. That was achieved by engineering the BiFC constructs with fragments of GFP (green fluorescent protein) fused with PAR-5 and H4. The occurrence of the interaction between the two proteins of interest was confirmed with the fluorescence observed in vivo.

Despite their numerous advantages, the BiFC method has some serious shortcomings that are to be considered. The most important one is that BiFC formation is irreversible. This can be useful in detecting weak interactions, but makes them useless in studying dynamic protein complexes [64]. On the other hand, BiFC probes based on fluorescent proteins are not optimal for long-term and/or single-molecule protein–protein interactions visualization because of their brightness and photostability, which are lower in comparison with organic dyes [65]. Another shortcoming is the temperature dependence as BiFC probes perform best at lower temperatures (25 °C or even 4 °C in some cases). This makes the approach unsuitable for some types of cells [66]. These and a number of other limitations should be taken into consideration when choosing BiFC probes for research.

#### 2.2.4. Reader Domain-Based Techniques

**Figure 5:**
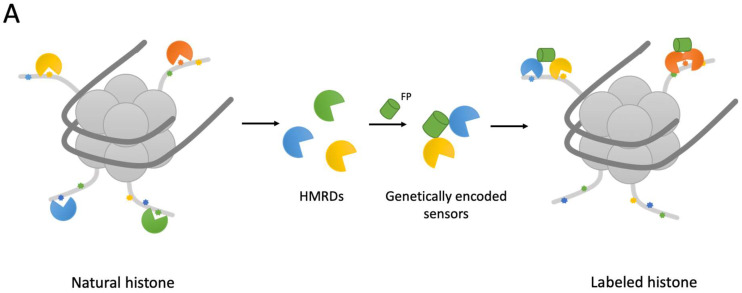

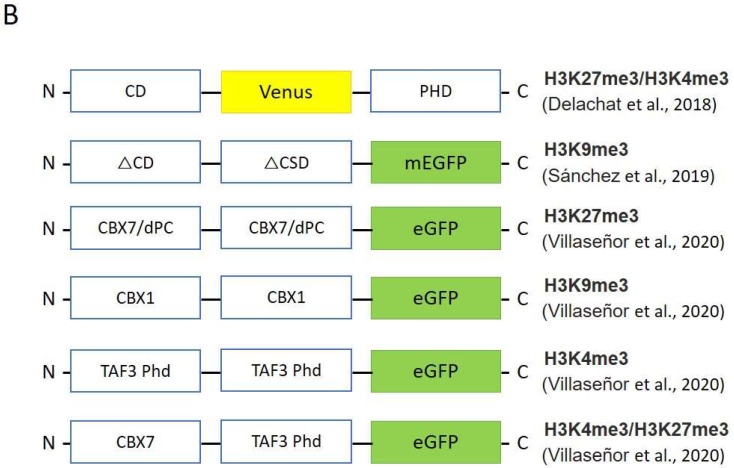
Reader domain-based sensors for live-cell imaging. (**A**) General design of reader domain-based sensors. (**B**) Domain structures of reporters described below [79,80,81].

Histone modification reader domains (HMRD) are key players in the interpretation of the histone code by binding to modified histones and attracting proteins with specialized functions required at a given chromatin locus [67]. Reader domains typically provide an accessible surface (such as a cavity or surface groove) to accommodate a modified histone residue and determine the modification (acetylation vs. methylation) or state specificity (such as mono- vs. trimethylation of lysine). PHD, chromo, tudor, PWWP, WD40, BAH, ADD, ankyrin repeat, MBT and zn-CW domains are some of the methyl-lysine-binding motifs found in the reader proteins, which can recognize target methyl-lysines, whereas BRD, YEATS, Yaf9, ENL, AF9, Taf14 and Sas5 prefer acetylated lysines [68]. The schematic representation of reader domain-based sensors observed in this paper is presented in Figure 5.

Firstly, a considerable number of scientific groups reported the crystal structures of reader domains, described their binding properties and determined their dissociation constants: for hMPP8 (MPHOSPH8) [69,70], ATRX-ADD [71], YEATS domain of AF9 [72], YEATS domain of Yaf9 [73], DPF3b [74], homeodomain of ING2 [75], chromodomain of CBX7 [76], etc. Mostly, these works were focused on the mechanism of histone modification recognition and the evaluation of additional proteins involved in reader complex formation. 

Then, Kungulovski et al. performed a proof-of-principle study, in which they characterized the specificity of several engineered histone modification-interacting domains (HMIDs) using the CelluSpots histone peptide arrays and compared them with ENCODE-validated antibodies for the same PTM [77]. It was shown that MPHOSPH8 Chromo and ATRX ADD HMIDs are comparable to ENCODE-validated antibodies according to their binding properties. Additionally, the authors investigated the applicative potential of HMIDs mentioned previously in ChIP-like experiments and demonstrated that the MPHOSPH8 Chromo and ATRX ADD CIDOP-qPCR profiles are highly comparable to the corresponding anti-H3K9me3 antibody.

After that, the idea that recombinant HMRDs can be used to create genetically encoded fluorescent sensors to monitor the activity of histone-modifying proteins in living cells was implemented. The same group, which previously described HMIDs, published a variant of fluorescent sensor with double specificity: it consisted of two fused recombinant histone modification-interacting domains (HiMIDs) for the direct detection of loci with doubly modified chromatin [78]. The authors fused the MPP8 chromodomain and DNMT3A PWWP domain, which have a binding specificity for H3K9me3 and H3K36me2/3, respectively, and demonstrated the new sensor’s specific interaction with H3K9me3–H3K36me2/3 doubly modified chromatin in comparison with CIDOP- and ChIP-seq data analysis.

In 2018, Delachat et al. developed chromatin-sensing multivalent probes (cMAPs) by combining two HMRDs with different specificities in one construct to visualize loci in which two different modifications coexist [79]. The authors used the chromodomain (CD) of the Polycomb (Pc) protein as a reader for H3K27me3, and plant homeodomain (PHD) originating from transcription initiation factor TFIID subunit 3 (TAF3) for H3K4me3. They showed specific localized clusters in the nuclei of living stem cells and confirmed the results with the ChIP assay and pull-down assay on synthetic bivalent nucleosomes.

Sanchez et al. also created the heterodimeric sensor visualizing H3K9me3 modification in living cells [80] (Figure 5B). The authors exploited HP1a chromodomain and chromo shadow domain as specifically binding units in their sensor that allowed them to evaluate changes in the distribution of the H3K9me3 mark in response to the environmental chemical atrazine (ATZ).

In 2020, Villaseñor et al. developed ChromID—a method for identifying the chromatin-dependent protein interactome [81]. The authors assembled the natural reader domains in a protein expression cassette with fluorescent protein; next, these engineered chromatin readers (eCRs) were expressed in living cells. It has been shown that HMRDs labeled with a fluorescent protein provide specific patterns in cell nuclei corresponding to loci enriched with one or another epigenetic modification. They used the following domains, chromodomains from CBX7 and Drosophila Polycomb for H3K27me3, CBX1 chromodomians for H3K9me3 and PHD domains from TAF3 for H3K4me3, and identified 58 high-confidence H3K9me3-associated proteins linked to pericentric or telomeric heterochromatin. In addition, Villaseñor and co-authors created the bivalent eCR (CBX7-TAF3-eCR), which ensured the combinatorial recognition of bivalent H3K4me3 and H3K27me3 loci and enabled the discovery of 33 high-confidence factors associated with bivalent chromatin. 

Summarizing, the reader domain-based workflow can be a powerful tool for our deep understanding of the functioning of distinct chromatin states and reader domains in gene regulatory mechanisms.

### 2.3. Super-Resolution Imaging

Our current understanding of higher-order chromatin structures defined by distinct histone modifications is derived indirectly from in vitro biochemical experiments, such as chromatin immunoprecipitation (ChIP), due to the poor resolution of traditional light microscopy [82,83] and chromatin conformation capture [84]. These assays frequently use fragmented DNA from pooled cell populations and thereby lose information at the single-cell level. Recent progress in super-resolution fluorescence microscopy now allows the imaging of chromatin structures below the diffraction-limited resolution in both fixed and live cells. There are three main types of super-resolution fluorescence microscopy techniques: structured illumination microscopy (SIM) [85], stimulated emission depletion microscopy (STED) [86] and single molecule localization microscopy (SMLM). The latter can directly visualize chromatin organization and chromatin states at a spatial resolution down to ~10 nm in single cells even in the spatial context of tissue with a minimal effect on biological samples [87]. Additionally, the ability to perform live-cell imaging based on SMLM makes it the most popular super-resolution technique for the visualization of chromatin architecture in situ in nanoscale at the single-cell level. For example, the combination of PALM [88] and single-nucleosome tracking allowed us to define higher-order structures and trace their dynamics in live mammalian cells [89. However, only a few works have been devoted to monitoring the state of at least one histone modification using super-resolution microscopy.

In the work by Conic et al., the authors were successful in high-resolution live-cell imaging. They offered the electroporation step for intracytoplasmic delivery labeled primary antibodies and Fabs into cultured cells and could image the dynamics of phosphorylated histone H2AX [90]. The authors developed a versatile antibody-based imaging approach (VANIMA) to monitor endogenous transcription factors and post-translational histone modifications allowing for the exact tracking of these targets in the 3D nucleus.

**Figure 6:**
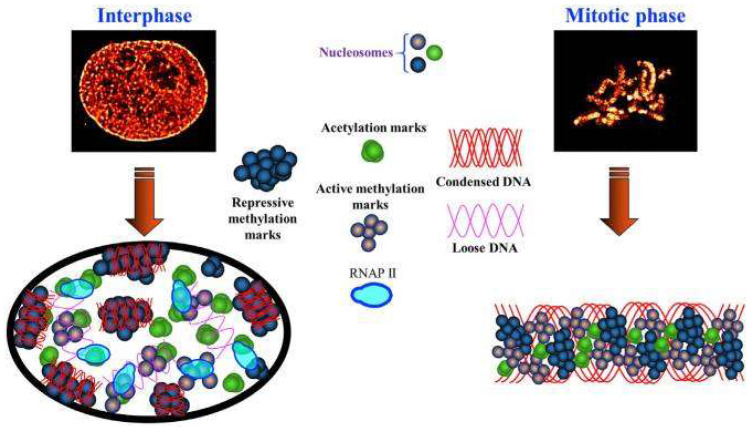
Model to illustrate the spatial organization of the chromatin environment at interphase and mitotic phases. Three distinct structural groups of active histone acetylation, active histone methylation, and repressive histone methylation, as well as their spatial relationship with active transcription machinery are shown. Modified from [91].

Other groups went further and established a goal to describe the detailed information of epigenetic modifications important for cellular functions and activities lacking in previous experimental designs. Xu, Liu et al. [91] used localization-based super-resolution microscopy and visualized the spatial relationship between histone marks and DNA compaction, and also described higher-order chromatin structures. They categorized them into three major types: (1) transcriptionally active histone acetylation marks that form spatially segregated nanoclusters; (2) transcriptionally active histone methylation marks that form spatially dispersed and heterogeneous nanodomains; and (3) transcriptionally repressive histone methylation marks that form highly condensed mega-sized clumps. Alexa Fluor 647 conjugated to the secondary antibodies was used for stochastic optical reconstruction microscopy (STORM) [92] imaging (Figure 6).

**Figure 7:**
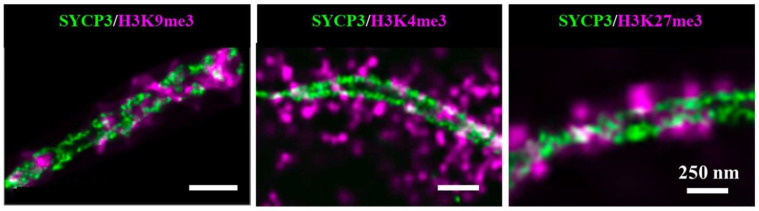
Visualizing spatial epigenomics using localization-based super-resolution microscopy. Two-color super-resolution images of chromosomes (green) and histone marks (magenta) via immunostained with anti-SYCP3 (Alexa 555) and anti-histone modifications (Alexa 488). Adapted with permission from [94], 2022, John Wiley and Sons.

Prakash et al. aimed to demonstrate the power of SMLM to describe the epigenetic landscape of pachytene chromosomes [93]. They revealed three distinct spatial morphologies in the chromosome structure of pachytene stage of meiosis prophase I: (i) radial chromatin identified by H3K4me3 indicative of actively transcribed chromatin, (ii) polar chromatin identified by H3K9me3 indicative of centromeric chromatin, and (iii) tangential chromatin identified by H3K27me3 indicative of repressed chromatin (Figure 7). 

In general, observing histone modifications at the single-molecule level can contribute to describe the roles of epigenetic modifications in higher-order genomic architecture.

## 3. Conclusions and Perspectives

Recent years have been characterized by a high interest in research at the level of single cells [95]. For example, single-cell transcriptomics became a popular tool to reveal previously hidden cell plasticity and heterogeneity in various models. The three-dimensional organization of genome and epigenetics are not an exception in this respect, attracting efforts to characterize it in individual nuclei [96,97,98]. Fluorescence microscopy is intrinsically focused on single-cell investigations. Thus, it represents an additional approach for the characterization of the epigenome at the single-cell level. The development of novel methods for the visualization of epigenetic modifications, especially in live cells, to study chromatin functioning and the high-throughput screening of epigenetically active compounds is a considerable challenge for scientists all over the world. 

Several methods mentioned above, such as MIEL [10], HiMIDs [78], cMAPs [79] and ChromID [81], offer a significant translational potential for the high-throughput screening of epigenetically active compounds. On the one hand, it could be a search for drugs inducing cancer cell differentiation, or rejuvenating drugs. On the other hand, fluorescence-based screening could be used as a safety test to reveal the hidden epigenetic toxicity of compounds that are under development or already in use [99]. However, the computation-rich MIEL strategy works with fixed cells, thus missing the dynamic picture of epigenetic remodeling. On the contrary, the published methods that employ genetically encoded probes for live-cell imaging do not attempt a precise mathematical characterization of images. We believe that the combination of MIEL with continuous imaging of genetically encoded fluorescent probes in live cells—LiveMIEL—would provide significant progress in the field. Indeed, LiveMIEL platform can potentially detect dynamic epigenetic changes in individual cells to give insights into the basic problems (e.g., cell differentiation or dedifferentiation in stem cell, developmental and regenerative biology) as well as to be applied for high-throughput screening.

The current implementation of MIEL and HMRD-based methods is limited by the light diffraction barrier: all fluorescent spots located closer than ~200 nm are indistinguishable from one another. It may be useful to extract more information from the epigenetic landscape using super-resolution fluorescence microscopy [100]. Moreover, some nanoscopy modalities are compatible with live-cell imaging [101]. Super-resolution techniques are capable of bringing the resolution limit down to 20 nm, enabling the visualization of key structural elements of chromatin organization such as individual nucleosomes. Moreover, a recently developed method MINFLUX, in which single molecules are tracked by a donut-shaped laser beam, enables the resolution of a few nanometers in 3D [102]. The application of MINFLUX can lead to a breakthrough in the understanding of the spatial organization of epigenetic marks in specific loci, as well as their dynamics in a variety of normal and pathological processes in living cells.

Another limitation of data obtained through the live imaging of histone marks is the lack of correspondence between the epigenetic landscape and the cell cycle phase in an individual cell. To gain such information, it may be useful to implement Fluorescent Ubiquitin-based Cell Cycle Indicator (FUCCI) [103,104].

A further promising direction is the application of phototoxic fluorescent proteins [105], such as KillerRed, miniSOG or their variants fused to HMRDs. It would enable the induction of strictly localized oxidative stress to damage chromatin nearby targeted epigenetic marks. Since light can be focused on a submicron region, particular loci can be probed with such a technique.

The most challenging task is to correlate intranuclear fluorescent patterns with sequence-based profiles of corresponding epigenetic modifications. Data for selected specific loci can be obtained by their co-labeling using established techniques such as fluorescence in situ hybridization (for fixed cells) or endonuclease-deficient Cas fused with fluorescent tag (for live or fixed cells) [106]. A large database of cell samples characterized in parallel by both fluorescent probes and ChIP-Seq could ultimately solve this problem.
